# Vaginal stones caused by urethrovaginal fistula: A case report

**DOI:** 10.1097/MD.0000000000018003

**Published:** 2019-11-22

**Authors:** Dongmei Wei, Yao Xie, Xiaoyu Niu

**Affiliations:** a Department of Gynecology and Obstetrics, West China Second Hospital, Sichuan University; b Key Laboratory of Birth Defects and Related Diseases of Women and Children (Sichuan University), Ministry of Education; c Department of Gynecology and Obstetrics, Sichuan Academy of Medical Sciences & Sichuan Provincial People's Hospital, China.

**Keywords:** congenital genitourinary malformations, urethra-vaginal fistula, urinary tract infection, vaginal calculus, vesicovaginal fistulas

## Abstract

**Rationale:**

Vaginal stone is a rare condition that is often misdiagnosed due to its low incidence. It can be divided into 2 types, primary vaginal and secondary vaginal, based on the etiology of the disease. Vaginal stones involve pathologic calcification. The formation of vaginal stones is mainly due to stasis and urine infection. We describe a procedure for the safe extraction of vaginal stones.

**Patient concerns:**

We report a case of a 25-year-old female patient with congenital genitourinary malformation and urethrovaginal fistula. A urogenital tract malformation repair operation was performed before she was 21 years old. Frequency of urination occurred before and after menstruation for 9 years, and dyspareunia occurred for 1 year.

**Diagnoses:**

B ultrasound examination showed a 59 × 55 × 23 mm fusiform region of increased signal intensity in the vagina followed by a sound shadow. We performed a gynecologic examination and found that the long diameter of the vaginal opening was 20 mm. A brown substance observed in her vagina had a hard texture and felt like a stone, and a palpation hand test revealed the size was approximately 60 × 50 mm. A cystoscope was inserted into the urethra and revealed that the broken end of the urethra was connected to the vagina. The proximal broken end of the urethra was 20 mm from the distal end.

**Interventions:**

The purpose of this operation was to make a definite diagnosis and remove the stones. We performed vaginal stone removal surgery and cystoscopy under anesthesia.

**Outcomes:**

We removed the stone successfully. The patient was discharged from the hospital after a smooth recovery without any complications. Follow-up was conducted 1 month after the operation and then every 3 months.

**Lessons:**

Although vaginal stones are rare, we must pay attention to this disease, especially in patients with congenital genitourinary malformations and urethrovaginal or vesicovaginal fistulas. Obstruction of urine discharge combined with repeated urinary tract infection is the main cause of vaginal stone formation. For these patients, follow-up every 3 months, including a physical examination, B-mode ultrasonography of the urinary system and cystoscopy if necessary, can avoid the occurrence of the disease.

## 1 Introduction

Vaginal stone is a rare condition that is often misdiagnosed due to its low incidence. It can be divided into 2 types, primary vaginal and secondary vaginal, based on the etiology of the disease. Primary vaginal stones are formed by the deposition of inorganic salts by stagnant urine within the vagina and are mainly caused by congenital genitourinary dysplasia,^[1]^ trauma, urethrovaginal fistula secondary to scars after gynecologic surgeries,^[2–4]^ ectopic ureters, and vaginal outlet obstruction,^[5,6]^ ultimately resulting in the formation of vaginal calculi due to the continuous deposition and infection of the urine in the vagina. Secondary vaginal stones are often attributed to the erosion of surgical meshes or other foreign bodies left in the vagina, with inorganic salts in the urine gradually depositing around the foreign bodies over time.^[7–9]^ In this article, we present the case of a patient with a primary vaginal stone that was caused by a urethrovaginal fistula. Follow-up was conducted 1 month after the operation and then every 3 months.

## 2 Case report

**Figure 1:**
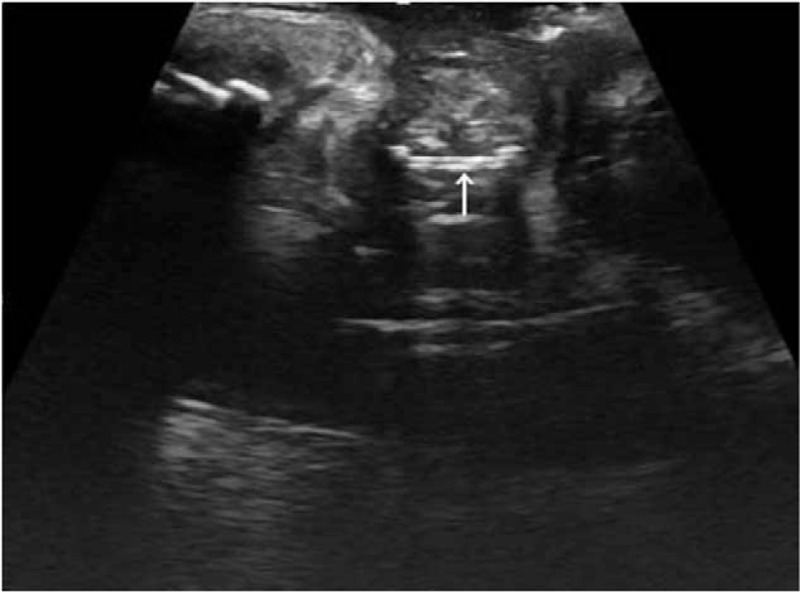
A transabdominal B ultrasound image showed a 59 × 55 × 23 mm fusiform region with increased signal intensity in the vagina followed by a sound shadow.

The patient was a 25-year-old female patient who was admitted to hospital on May 28, 2018 for “9 years of frequent urination before and after menstruation and 1 year of dyspareunia.” When the patient was four years old, she had uncontrollable and persistent urine leakage, for which she had been diagnosed with “congenital bladder valgus and urethral mucosal prolapse” and underwent surgery (a urogenital tract malformation repair operation). After the surgery, her persistent urine leakage remained uncontrolled. The patient gained control over her urination approximately 2 to 3 years after the surgery, when her urine overflowed slowly without spray urination. Occasionally, the patient presented with spray urination during urinary urgencies; however, no involuntary urine overflow was noted. Since the patient's menarche at 17 years of age, the number of urinations had increased 4 to 5 days before and after menstruation. The urinary volume was small, and sometimes her urinations occurred as frequently as once per hour. Five years ago, the patient presented with repeated urinary tract infections that usually lasted for 6 months before they were resolved by anti-infection treatment. A year ago, when the patient started having sex with her boyfriend, the man's penis bled due to abrasion, and the patient palpated a hard lump in her vagina. The patient visited a local hospital, and a B-ultrasound examination showed a 59 × 55 × 23 mm fusiform region of increased signal intensity in the vagina followed by a sound shadow. There were no obvious abnormalities in the uterine attachments (Fig. 1).

**Figure 2:**
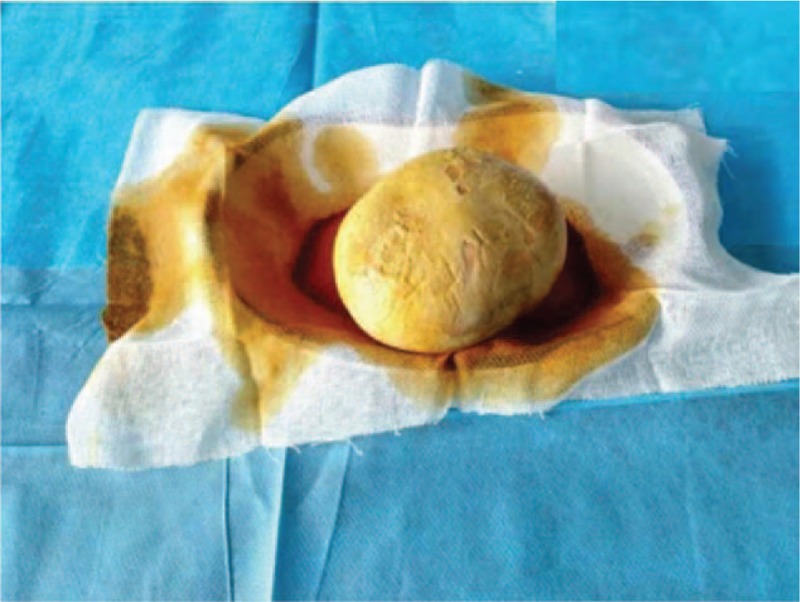
Image of the vaginal stone removed during surgery.

A physical examination after admission revealed that her vulva shape was normal. There was an old longitudinal surgical scar in the middle of her pubic symphysis, and its lower end reached the left side of her clitoris. A urethral catheter was inserted into the urethra through the opening for approximately 70 mm, and no urine flow was observed. The long diameter of the vaginal opening was 20 mm. A brown substance seen in her vagina had a hard texture, felt like a stone, was visible on direct observation, and measured approximately 60 × 50 mm in size. There was urine outflow during the palpation. The urinary fistula and the cervix could not be seen. After admission, while under anesthesia, vaginal stone removal and cystoscopy were performed. The tissues around the fistula were carefully separated, and the stone was completely removed (Fig. 2). At 3 months after the stone was removed, we repaired the urethrovaginal fistula. An examination revealed that the cervix was normal. The cystoscope was inserted into the urethra from the urethral opening for 20 mm and revealed that the broken end of the urethra was connected to the vagina. The proximal broken end of the urethra was 20 mm from the distal end. The surface of the fistula was mucosal, and the bladder was seen after the cystoscope entered the broken end of the urethra. No obvious abnormality was observed in the bladder cavity, and urine ejection was observed at the opening of the bilateral ureters. The patient was diagnosed with a vaginal stone and urethrovaginal fistula. The patient had recovered well at 1 month after the operation. Urine frequency symptoms and pain during sexual intercourse were relieved. A follow-up was conducted 1 month after the operation and then every 3 months.

Because this report only reviewed previous data and did not involve any human trials, there was no need to conduct special ethical review, and ethical approval was not necessary. Written informed consent was obtained from the patient for the publication of this case report and its accompanying images.

## 3 Discussion

Vaginal stones are a pathologic calcification disease, and the formation of vaginal stones is mainly caused by stagnant urine and infections. Stagnant urine inside the vagina facilitates urease-producing bacterial infections (such as *Klebsiella* or *Escherichia coli*), so that the acidic environment of the vagina becomes alkaline, which contributes to the formation of triphosphate crystal stones, and triphosphate is the most common vaginal stone component known to date.^[10]^ Despite the lack of medical history in the patient, who was young in age, it can be inferred from the limited data provided by the patient that she had congenital urinary system malformation, and the urethrovaginal fistula was caused by surgical failure in childhood. Long-term urine stagnation in the vagina and repeated infection, when combined with a vaginal opening covered by the hymen, resulted in the accumulation of uterine and vaginal secretions, with the calcium salts in the urine easily depositing and forming primary vaginal stones.

The formation of vaginal stones is a slow process, and most cases are only discovered when the vaginal stones are large enough to cause obvious clinical symptoms. All urogenital stones, including vaginal stones, can cause urinary tract irritation symptoms, such as frequent urination and urination urgency. Vaginal stones can also be accompanied by dysuria, vaginal pain, dyspareunia, partner complaint of pain during sex, etc.^[11–13]^ This patient presented with frequent urination, urination urgency, and bleeding from her partner's penis due to abrasion during sex. As such, the diagnosis of vaginal stones should be considered. If the vaginal stone is pressing toward the rectum, it can cause a desire to defecate, stool deformation, and anal pain during sitting. Vaginal stones are often complicated with symptoms of infection, such as fever, as well as increased vaginal secretions, and unpleasant odor.

This patient has had repeated urinary tract infections. Because of the low incidence of the disease, vaginal stones often do not raise enough attention clinically. Especially in patients with concurrent infections, there are often nonspecific infection symptoms, and this makes it difficult to distinguish this disease from nephritis, urinary tract infections, etc. Often, more emphasis is placed on the treatment of the infection, and a gynecologic examination is neglected, resulting in long-term infection control failure. This also leads to missed diagnoses and the misdiagnoses of vaginal stones. Therefore, gynecologic examinations provide a relatively reliable means for the diagnosis of vaginal stone. Even if a young girl has symptoms including frequent urination and urination urgency, a reproductive system examination should not be neglected, and an anal examination should be performed when necessary. B ultrasound and X-ray examinations of the pelvis are also helpful. Ultrasonography can confirm the diagnosis of stones and show the location of the stones and their relationship with the pelvic organs.^[6]^ When the stone is large and difficult to identify, it is feasible to perform pelvic computed tomography and magnetic resonance imaging (MRI). Pelvic MRI can clearly show the structure of the endometrium and muscle layers as well as the exact position of the stone.^[12]^ This patient was diagnosed using a B-ultrasound examination and gynecologic examination, which showed a region of high signal intensity and a structure with a rock-hard touch during palpation.

Surgery is the preferred treatment for vaginal stones. The surgical methods include open surgery, stone fragmentation and extracorporeal shock wave lithotripsy, and endoscopic intervention.^[14]^ The best treatment regimen should be decided based on the patient's specific conditions and available equipment. During the stone removal process, any adhesion between the stone and the surrounding tissues should be carefully separated to prevent damage to the surrounding tissues (the bladder, rectum, etc). For patients with a urethrovaginal or vesicovaginal fistula, due to severe edema of the tissues around the stone, it is not advisable to perform fistula repair while removing the stone. The fistula should be repaired 3 to 6 months after stone removal when the edema around the fistula has subsided.^[15]^ For older patients or patients with other serious complications, a cystostomy can be selected. After the stone is removed, early diagnosis and treatment should be carried out for urinary tract malformation, which can cause vaginal stones. Patients should pay attention to personal hygiene and personal care and avoid urine stagnation in the vagina to prevent infections. Early removal of a vaginal opening obstruction can effectively prevent the formation of vaginal stones. This patient developed a urethrovaginal fistula, and a fistula should be actively repaired 3 to 6 months after the removal of the stone to facilitate the gradual recovery of bladder function and achieve autonomous urination.

This case report aims to remind medical practitioners that although vaginal stones are rare, we must pay attention to this disease, especially in patients with congenital genitourinary malformations and urethrovaginal or vesicovaginal fistulas. For these patients, follow-up should be performed every month, and the timely control of urinary tract infections can avoid the occurrence of the disease.

## Author contributions

**Conceptualization:** Dongmei Wei, Xiaoyu Niu.

**Data curation:** Dongmei Wei.

**Formal analysis:** Dongmei Wei.

**Investigation:** Yao Xie.

**Methodology:** Yao Xie.

**Project administration:** Yao Xie.

**Resources:** Yao Xie.

**Software:** Yao Xie.

**Supervision:** Yao Xie.

**Validation:** Yao Xie.

**Visualization:** Yao Xie.

**Writing – original draft:** Dongmei Wei.

**Writing – review & editing:** Xiaoyu Niu.
